# A Novel TCR Transgenic Model Reveals That Negative Selection Involves an Immediate, Bim-Dependent Pathway and a Delayed, Bim-Independent Pathway

**DOI:** 10.1371/journal.pone.0008675

**Published:** 2010-01-13

**Authors:** Damian Kovalovsky, Mark Pezzano, Benjamin D. Ortiz, Derek B. Sant'Angelo

**Affiliations:** 1 Immunology Program, Sloan-Kettering Institute, Memorial Sloan-Kettering Cancer Center, New York, New York, United States of America; 2 Department of Biology, The City College of New York, New York, New York, United States of America; 3 Department of Biological Sciences, City University of New York, Hunter College, New York, New York, United States of America; 4 Louis V. Gerstner Jr. Graduate School of Biomedical Sciences, Memorial Sloan-Kettering Cancer Center, New York, New York, United States of America; 5 Weill Graduate School of Medical Sciences of Cornell University, New York, New York, United States of America; New York University, United States of America

## Abstract

A complete understanding of negative selection has been elusive due to the rapid apoptosis and clearance of thymocytes in vivo. We report a TCR transgenic model in which expression of the TCR during differentiation occurs only after V(D)J-like recombination. TCR expression from this transgene closely mimics expression of the endogenous TCRα locus allowing for development that is similar to wild type thymocytes. This model allowed us to characterize the phenotypic changes that occurred after TCR-mediated signaling in self-reactive thymocytes prior to their deletion in a highly physiological setting. Self-reactive thymocytes were identified as being immature, activated and CD4^lo^CD8^lo^. These cells had upregulated markers of negative selection and were apoptotic. Elimination of Bim reduced the apoptosis of self-reactive thymocytes, but it did not rescue their differentiation and the cells remained at the immature CD4^lo^CD8^lo^ stage of development. These cells upregulate Nur77 and do not contribute to the peripheral T cell repertoire in vivo. Remarkably, development past the CD4^lo^CD8^lo^ stage was possible once the cells were removed from the negatively selecting thymic environment. In vitro development of these cells occurred despite their maintenance of high intracellular levels of Nur77. Therefore, in vivo, negatively selected Bim-deficient thymocytes are eliminated after prolonged developmental arrest via a Bim-independent pathway that is dependent on the thymic microenvironment. These data newly reveal a layering of immediate, Bim-dependent, and delayed Bim-independent pathways that both contribute to elimination of self-reactive thymocytes in vivo.

## Introduction

The establishment of a mature T cell repertoire that is able to recognize foreign antigens without overt self-reactivity is established in the thymus through a process termed “negative selection” or “clonal deletion”. After “in-frame” V(D)J recombination of the T cell receptor (TCR)α locus, the newly rearranged TCRα pairs with the TCRβ forming a mature TCR. Interactions of the TCR with rare endogenous peptides presented by cortical thymic epithelial cells in the context of MHC molecules leads to the differentiation of thymocytes and migration to the medulla. Aire-expressing medullary thymic epithelial cells that express tissue specific antigens and dendritic cells located in the cortico-medullary boundary recognize and delete thymocytes expressing TCRs with overtly self-reactive specificities [1], [2]. Negative selection has also been observed in the thymic cortex [3], [4]. Although TCR transgenic mice against the male H-Y antigen have shown that clonal deletion can happen at a late double negative (DN) or early double positive (DP) stage, lack of deletion of DP thymocytes in a mouse model that first expresses the H-Y TCR in DP thymocytes suggested that deletion occurs during the differentiation to the single positive (SP) stage of development [5]. Therefore, the time and developmental stage at which negative selection takes place is still matter of debate [6].

TCR transgenic mice have been important for the study of T cell selection in the thymus. In particular, because the analysis of differentiation without the variability conferred by the polyclonal T cell repertoire is possible. However, a complete understanding of the mechanism of negative selection has been elusive due to the rapid apoptosis and clearance of thymocytes *in vivo*. Also, the abnormally high level of TCR expression already at the DN3 stage in TCR transgenic mice causes aberrant development including differentiation towards DN and CD8αα intraepithelial lymphocytes that have characteristics of γδ T cells [5], [7]. This lineage diversion bypasses the DP stage. Even mice generated by nuclear transfer using DNA with a rearranged TCR from a mature T cell have aberrant thymocyte development. Therefore, disrupted T cell development occurs even with the endogenous TCRα locus if it has already undergone rearrangement [8]. These data suggest that normal expression of the TCR during differentiation is controlled by V(D)J recombination events in addition to transcription.

Despite the difficulties of studying negative selection *in vivo*, considerable progress has been made towards the understanding of the signaling cascades that are essential for apoptosis of DP thymocytes triggered by strong TCR signals [9]. For example, increased phosphorylation of Jnk [10], and upregulation of Nur77 and Bim have been shown to be essential for apoptosis of thymocytes during negative selection [11], [12].

Here we study the fate of self-reactive thymocytes in mice carrying a recombination-dependent TCR transgene. Expression of a functional TCRα chain from this transgene is dependent upon a V(D)J-like recombination event, closely mimicking the requirements of the endogenous locus. Using this new mouse model, we identified self-reactive thymocytes with an immature CD4^lo^CD8^lo^ phenotype. Most of these cells were undergoing apoptosis. To study the mechanisms of negative selection, the TCR transgene was crossed to Bim-deficient mice. As expected, the frequency of apoptotic thymocytes was significantly reduced. Bim-deficient self-reactive thymocytes did not differentiate to the CD8 SP stage, but rather maintained the CD4^lo^CD8^lo^ phenotype. However, CD4^lo^CD8^lo^ thymocytes were able to initiate CD8 differentiation upon release from the negative selecting environment by transfer to *in vitro* systems. Our data supports a model of negative selection that involves Bim-dependent and -independent pathways and indicates that chronic self-reactive TCR signals are necessary for the Bim-independent deletion of thymocytes.

## Results

### Thymocyte Development in Rec-HY Mice

**Figure 1:**
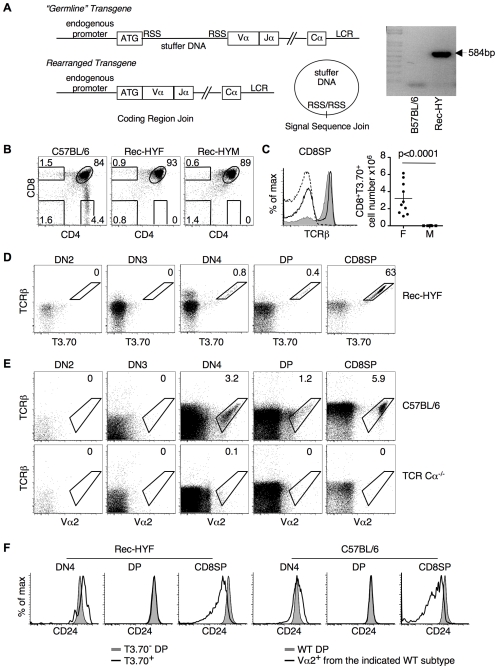
TCR expression in thymocytes from Rec-HY transgenic mice. **A**) Diagram representing the Rec-HY transgene. In the “Germline” conformation, the start ATG codon was separated by stuffer DNA from the rest of the coding sequence for the TCR-α subunit specific for recognition of the male HY antigen. This “stuffer” DNA has recombination signal sequences (RSS) that are recognized by the V(D)J recombination machinery during the differentiation of thymocytes. Recombination leads to the “Rearranged” transgene which as result of TdT and DNA exonucleases activities is expected to give rise to both productive (in-frame) and non-productive (out-of-frame) products. Recombination also generates an excision circle, which is formed by the ligation of RSS ends. PCR analysis was used to identify the formation of the excision circle corresponding to the stuffer DNA in C57BL/6 and Rec-HY thymocytes. **B**) FACS analysis showing CD4 vs CD8 of total thymocytes from C57BL/6, Rec-HY (Rec-HY transgenic, HY TCRβ, TCR Cα^−/−^) female (Rec-HYF) and male (Rec-HYM) mice. The numbers indicate the percentage of cells in each quadrant. **C**) FACS analysis comparing membrane TCRβ levels in CD8 SP gate shown in panel (B) from Rec-HY females (solid line), males (dashed line), and C57BL/6 wild type mice (filled histogram). Analysis of T3.70^+^CD8 SP cell numbers in Rec-HY females (F) and males (M), horizontal and vertical bars represent the average and SEM respectively (Average F  = 5×10^6^, M  = 1×10^5^). **D**) FACS analysis showing the clonotypic HY-TCR (T3.70) and TCRβ levels in thymocytes at different differentiation stages from Rec-HY female mice. The numbers indicate the percentage of cells in each gate. **E**) FACS analysis of TCRα (Vα2) vs TCRβ in different thymocyte subpopulations found in C57BL/6 mice. Thymocytes from TCR Cα-deficient (TCR Cα^−/−^) mice are included to confirm the specificity of the staining. The numbers represent the percentage of events within the gate. D and E correspond to different experiments in which different antibodies (conjugated with different fluorochromes) against TCRβ were used. **F**) FACS analysis showing the CD24 levels on T3.70^+^ Rec-HY thymocytes from gates in (D), in comparison with DP thymocytes not expressing the HY-TCR (T3.70^−^) from the same mouse. CD24 levels on wild type (C57BL/6) Vα2+ thymocytes at different developmental stages from gates in (E) in comparison with total WT DP thymocytes are also represented. These results are representative of four independent experiments.

Selection of thymocytes was studied using a new recombination-dependent H-Y TCR transgenic model (Rec-HY). The H-Y TCR confers reactivity to the H-Y male antigen and, as a consequence, thymocytes expressing this TCR are negatively selected in males, but positively selected in females [13]. The Rec-HY transgene was constructed with the promoter and ATG start codon of the H-Y TCRα gene separated from the V-J coding sequence by a 5KB “stuffer” DNA fragment. The stuffer DNA fragment was flanked by recombination signal sequences (RSS) (**Figure 1A**). The split-gene arrangement cannot encode a functional protein in the unrearranged “germline” configuration. During thymocyte development the transgene was designed to undergo V(D)J-like somatic recombination, during which the stuffer fragment was expected to be removed and the promoter/ATG start codon were expected to fuse to the V-J region. The coding join ends were expected to be substrates for both TdT and DNA exonucleases, similar to endogenous V(D)J coding region joins. Therefore, many of the TCRα genes would not be in-frame and would not produce a functional TCR. The stuffer DNA fragment should be excised and the RSS sites ligated to form an excision circle. In the cells in which recombination produced an in-frame TCRα, the protein was expected to be first expressed at a point in development similar to endogenous TCRα chains.

This construct was cloned into a modified version of the ∼20 kb pTαCass, which contains the entire TCR Cα region [14]. The most significant modification of the pTαCass was the addition of the full TCRα locus control region (LCR) [15], which we found was substantially truncated in the pTαCass transgene vector. By breeding, the Rec-HY transgene was introduced into TCR Cα deficient mice that also carried a transgene encoding the H-Y TCRβ chain [13].

As expected, thymocytes that did not express an αβTCR, due to out of frame joins, behaved similarly to thymocytes in TCR Cα deficient mice and did not develop past the pre-selected DP stage (data not shown). This feature of the transgene resulted in a large population of DP thymocytes and, as a consequence, both female and male mice carrying the Rec-HY and H-Y TCRβ transgenes (Rec-HY) were found to have wild-type numbers of total thymocytes (data not shown). This is in sharp contrast to conventional H-Y αβ TCR transgenic mice, which have a 25–50% reduction of thymocytes in females and a >95% reduction in male mice [6], [16]. At the genetic level, the excision circle expected as a product of V(D)J-like recombination of the Rec-HY transgene was readily detectable by PCR in transgenic mice, but not in wild-type mice (**Figure 1A**).

As anticipated, selection of thymocytes expressing the MHC class I restricted H-Y TCR was skewed towards the CD8 lineage (**Figure 1B**). The levels of TCRβ expression in CD8 SP thymocytes from Rec-HY female mice were similar to those from wild type mice (**Figure 1C**). However, Rec-HY CD8 SP thymocytes had a higher proportion of cells not expressing an αβTCR. These cells are also found in TCR Cα deficient mice (data not shown), and correspond to intermediate SP (ISP) thymocytes that are differentiating to the pre-selected DP stage [17], [18]. CD8 SP thymocytes found in Rec-HY males were almost exclusively ISP thymocytes as shown by their lack of mature TCR (**Figure 1C**).

The clonotypic monoclonal antibody, T3.70, which is specific for the CDR3α region of the H-Y TCR, was used to determine when functionally rearranged H-Y TCRs were expressed in the Rec-HY mice. Analysis of T3.70^+^ CD8 SP thymocyte numbers showed a significant reduction in Rec-HY males as compared to females (**Figure 1C**). The first detected TCR expression in Rec-HY mice was at the DN4 stage of development. T3.70^+^ cells were also detected within the DP and CD8 compartments (**Figure 1D**). This pattern of T3.70^+^ TCR expression correlated with that of Vα2 expression in wild type mice. The specificity of the TCR staining was confirmed by analysis of TCR expression in TCR Cα-deficient mice (**Figure 1E**). T3.70^+^ DN4 thymocytes from Rec-HY mice and Vα2^+^ DN4 thymocytes from wild type mice similarly expressed high levels of CD24, ∼2-fold higher than DP cells, (**Figure 1F**), which is characteristic of DN cells. Previous reports have shown that the TCRα chain can be detected in DN thymocytes in wild type mice [19]–[22], and transfer of TCR^+^DN4 cells to fetal thymic organ cultures (FTOCs) gave rise to αβTCR lineages [22]. The percentage of Vα2^+^ DN4 thymocytes that we detected in wild type mice is higher than previously described [22]. This difference is most probably due to the stringent exclusion of lineage-differentiated cells that we performed (see materials and methods). The different intensities observed for TCRβ staining between panels 1D and 1E are due to the different fluorochromes used for the different experiments.

During the differentiation of DN thymocytes to the DP and SP stages CD24 levels are continuously reduced [23]. Similarly to wild type mice, differentiating T3.70+ thymocytes in Rec-HY females downregulated CD24 levels as the progressed to the DP and CD8 SP stages (**Figure 1F**), revealing the similarity to the differentiation of wild type thymocytes.

Overall, the Rec-HY undergoes V(D)J-like recombination and expresses the TCR similarly to the wild-type locus. Most importantly, the development of TCR transgenic thymocytes closely mimics wild type T cell development. These characteristics, as well as the presence of a pre-selected DP population, make this model an excellent tool to study thymocyte differentiation.

### H-Y TCR Expressing Thymocytes in Male Rec-HY Mice

**Figure 2:**
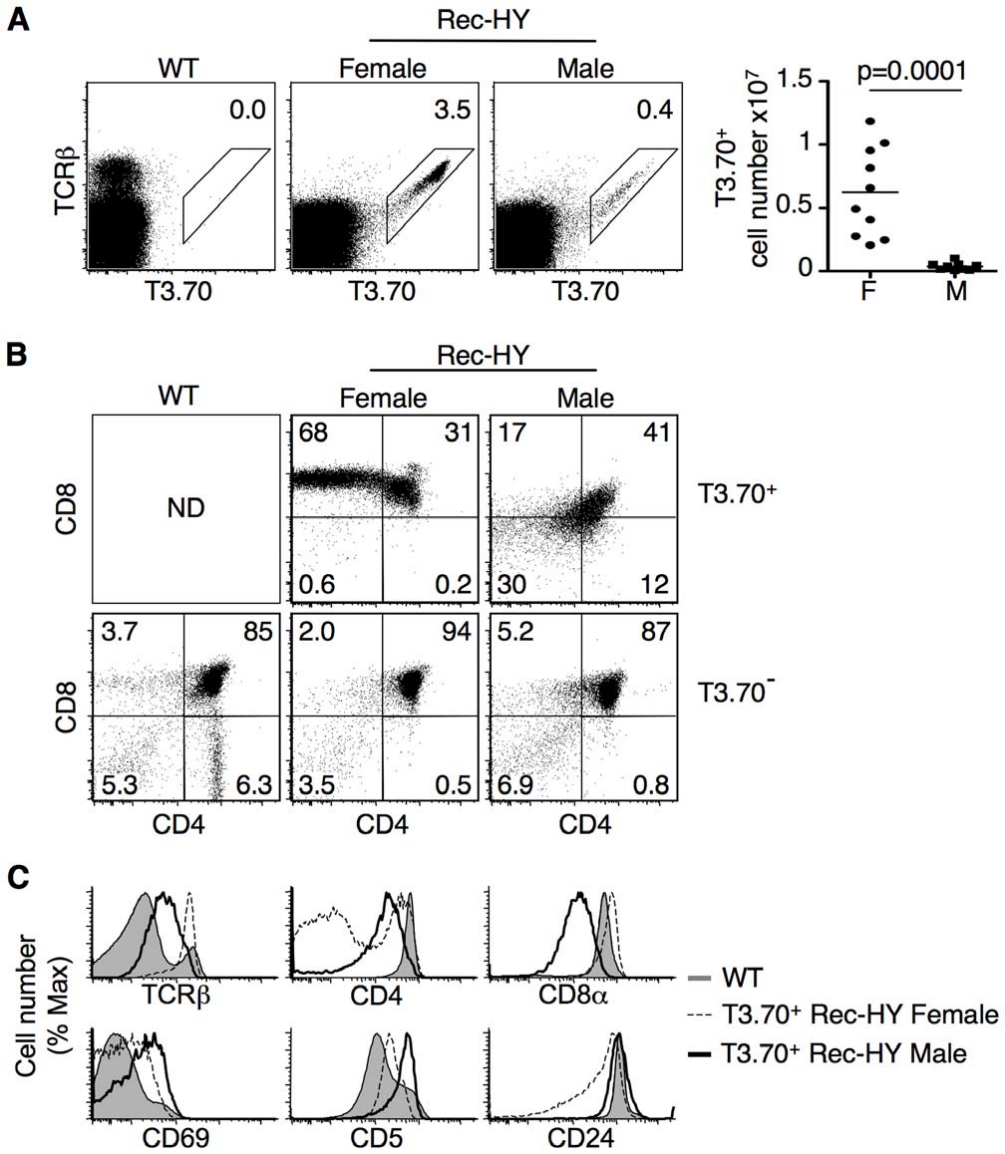
Differentiation of clonotypic thymocytes in Rec-HY transgenic mice. **A**) FACS analysis showing the membrane clonotypic HY-TCR (T3.70) and TCRβ in thymocytes from C57BL/6 (WT), Rec-HY female and male transgenic mice. The numbers represent the percentage of events within the T3.70 positive gate. Thymocyte numbers from Rec-HY females (F) and males (M) that express the HY-TCR (T3.70^+^) (n = 10, p = 0.0001). **B**) FACS analysis showing membrane CD4 vs CD8 from T3.70^+^ and T3.70^−^ thymocytes from C57BL/6 (WT) and Rec-HY female and male mice. The numbers represent the percentage of events within each quadrant. **C**) FACS analysis of surface markers on C57BL/6 (WT) thymocytes in comparison with T3.70^+^ thymocytes from Rec-HY female and male mice. (A and B) are representative of ten independent experiments, (C) is representative of four.

T3.70^+^ thymocytes were detected in female and, at substantially reduced numbers, in male Rec-HY mice (**Figure 2A**). Thymocytes expressing the H-Y TCR in males and females, however, had dramatically different phenotypes. H-Y TCR expressing thymocytes in female Rec-HY mice were found within the DP population and showed clear differentiation towards the CD8 SP stage of development (**Figure 2B**). In contrast, the T3.70^+^ thymocytes in male Rec-HY mice failed to differentiate towards the CD4 or CD8 lineages, but rather acquired a CD4^lo^CD8^lo^ phenotype (**Figure 2B**). Although this phenotype appears similar to the post-positive selection “double dull” population that we, and others, have described, the CD4 and CD8 levels were much lower than previously reported [24], [25].

T3.70^+^ cells from female mice had TCR, CD69 and CD5 levels consistent with that found on wild-type, post-selected thymocytes (**Figure 2C**). Furthermore, CD4 and CD24 levels were found to be downregulated as cells differentiated towards the CD8 SP stage. The phenotype of the T3.70^+^ thymocytes in males, in contrast, clearly indicated that their development was altered. For example, males displayed a distinct downregulation of TCR, CD4 and CD8 levels (**Figure 2C**). There also was an increase of CD69 and CD5 expression in comparison to positively selected thymocytes in Rec-HY female mice. These differences were consistent with T3.70^+^ cells in male mice receiving stronger TCR-mediated signals than the cells in female mice. Male T3.70^+^ thymocytes expressed high levels of CD24, which together with the CD4^lo^CD8^lo^ phenotype suggested that they were immature. Similar to the bulk of the male T3.70+ thymocyctes, CD8^lo^ cells in Rec-HY males were also CD24^hi^, TCRβ^lo^, and CD5^hi^. These cells were however CD69^lo^, which may facilitate their escape to the periphery [26], [27] (**Figure S1**).

### T3.70^+^ Rec-HY Thymocytes from Male Mice are Apoptotic

**Figure 3:**
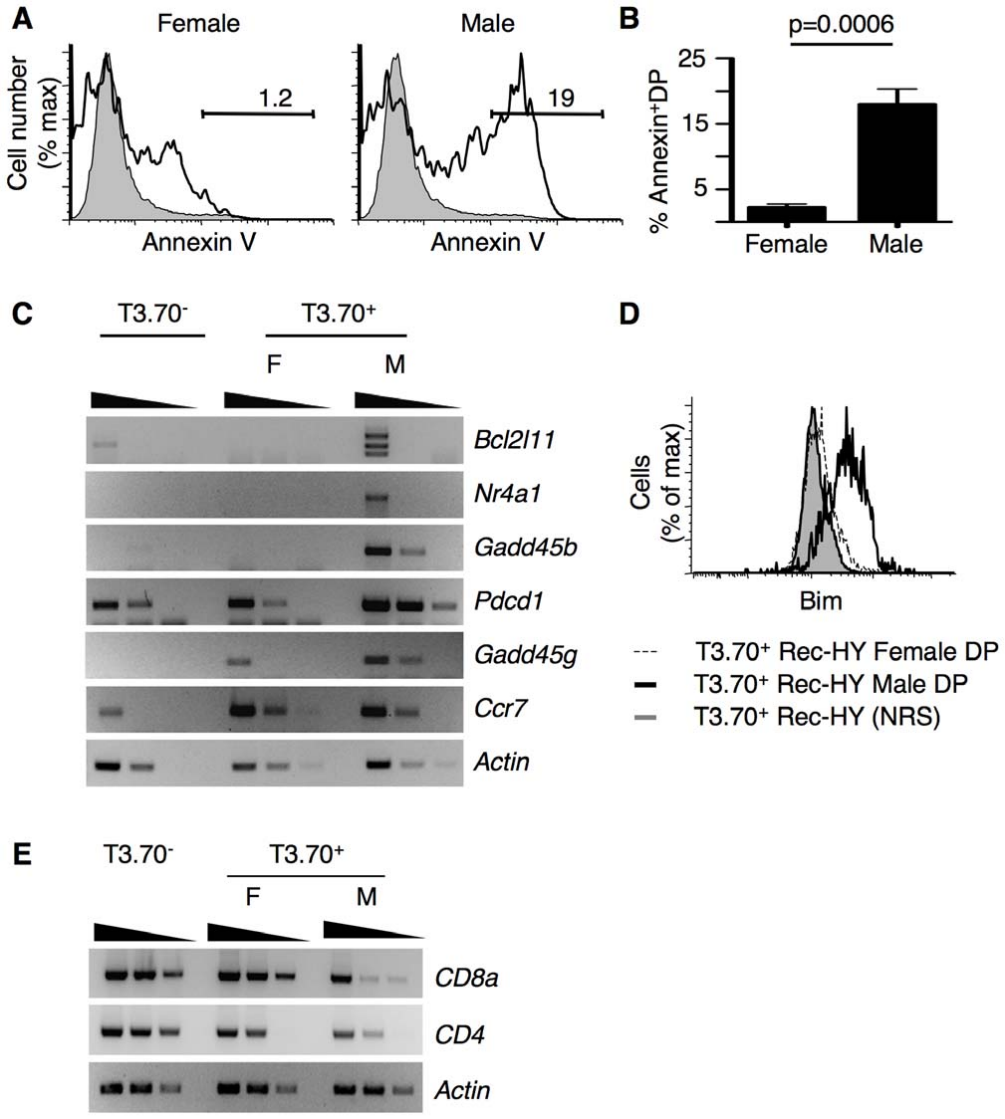
Clonotypic thymocytes from Rec-HY males are apoptotic. **A**) FACS analysis of annexin V staining on electronically gated DP thymocytes from Rec-HY females or males expressing the HY-TCR (T3.70^+^). The gray histogram represents the annexin V levels of T3.70^−^ pre-selected DP thymocytes. The numbers represent the percentage of events within the positive gate. **B**) Quantification of the percentage of apoptotic DP thymocytes expressing the HY-TCR (T3.70^+^) from Rec-HY females and males (mean ± SEM, n = 4, p = 0.0006). **C**) Semiquantitative RT-PCR (1∶5 serial dilution of DNA) of sorted T3.70^+^ DP thymocytes from Rec-HY transgenic mice and analysis of expression of genes associated with negative selection. T3.70^−^ corresponds to DP thymocytes not expressing the HY-TCR. T3.70^+^ F and M correspond to DP thymocytes expressing the HY-TCR from Rec-HY females and males respectively. **D**) Intracellular FACS analysis for Bim proteins in T3.70^+^ DP thymocytes from Rec-HY females and males. The gray histogram corresponds to staining with normal rabbit serum as first antibody. **E**) RT-PCR analysis (1∶5 serial dilution of DNA) for CD4 and CD8a expression in T3.70^+^ DP thymocytes sorted from Rec-HY female and male mice in comparison with T3.70^−^ DP thymocytes. (A and B) are representative of four independent experiments. (C, D and E) are representative of two.

The percentage of apoptotic thymocytes in male and female Rec-HY mice was determined by Annexin V staining. There was more than a 10-fold increase in the frequency of apoptotic cells among T3.70^+^ thymocytes from male as compared to female mice (**Figure 3** **A, B**). T3.70^+^ thymocytes in males also had higher mRNA expression levels of *Bcl2l11* (*Bim)*, *Nr4a1* (Nur77), *Gadd45b* and *Pdcd1* (Pd1) (**Figure 3C**). Increased expression of these genes has been shown during negative selection [28], [29]. Increased *Gadd45g* and *Ccr7* mRNA levels were also found in T3.70^+^ thymocytes from both male and female mice as compared to pre-selected T3.70^−^ DP thymocytes (**Figure 3C**), consistent with these cells having received TCR-mediated signals [30]. Self-reactive thymocytes also had elevated amounts of intracellular Bim (**Figure 3D**). Finally, the downregulation of CD4 and CD8 identified by FACS in Rec-HY male mice (Figure 2) was a consequence of decreased levels of CD8a and CD4 message (**Figure 3E**). Collectively our results show that T3.70^+^ thymocytes in Rec-HY male mice had the characteristics of immature CD4^lo^CD8^lo^ thymocytes that have received TCR signals and were apoptotic.

**Figure 4:**
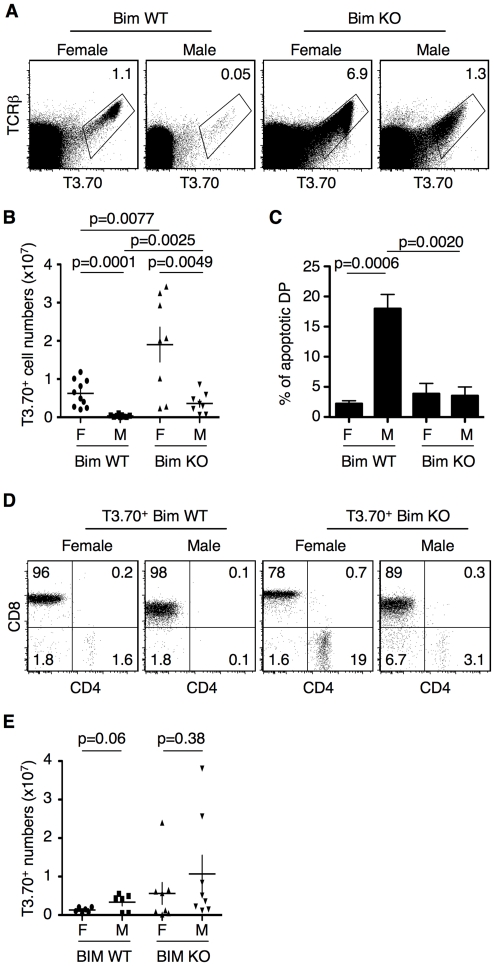
Bim-deficiency rescues the high apoptosis of T3.70^+^ thymocytes in Rec-HY. **A**) FACS analysis of thymocytes from Rec-HY transgenic males and females in Bim-sufficient (Bim WT) and Bim-deficient (Bim KO) backgrounds. The gates and numbers represent the percentage of clonotypic (T3.70^+^) thymocytes. **B**) T3.70^+^ thymocyte numbers from Rec-HY females (F) and males (M) in Bim-sufficient (Bim WT) or Bim-deficient (Bim KO) backgrounds. (Mean ± SEM, n≥8, the p values are indicated in the graph). **C**) Quantification of the percentage of apoptotic T3.70^+^ DP thymocytes in Rec-HY females and males in Bim-sufficient (Bim WT) or Bim-deficient (Bim KO) backgrounds (Mean ± SEM, n = 4 the p values are indicated in the graph). The data corresponding to the Rec-HY Bim-sufficient T3.70^+^ thymocyte numbers and the percentage of apoptotic T3.70^+^ DP thymocytes were shown in figures 2 and 3, respectively. The same data is shown again to allow for comparison with the Rec-HY Bim-deficient strains. **D**) FACS analysis of CD4 vs CD8 expression on electronically gated T3.70^+^ lymphocytes from Rec-HY Bim-sufficient and –deficient female and male spleens. The numbers represent the percentage of events within each quadrant. **E**) T3.70^+^ lymphocyte numbers in the spleens of the strains represented in D. The p values are represented within the graph. No significant differences were found. (A and B) are representative of eight independent experiments and (C) is representative of four, (D) is representative of six.

Bim has been shown to mediate apoptosis during the negative selection of DP thymocytes [12], [31], [32]. The importance of the expression of this protein was also suggested by its upregulation in T3.70^+^ thymocytes from Rec-HY male mice (Figure 3D). Therefore, we generated Rec-HYα, H-Y TCRβ, TCR Cα-deficient, Bim-deficient mice (Rec-HY Bim-deficient) by breeding, in order to evaluate the role of Bim during negative selection in our model. Increased numbers of T3.70^+^ thymocytes were observed in both male and female Rec-HY Bim-deficient mice relative to Rec-HY Bim-sufficient mice (**Figure 4A and B**). The increase in T3.70^+^ cells was clearly more pronounced in male mice (∼20-fold) than in females (∼6-fold). Despite the increase of T3.70+ cell numbers in Rec-HY Bim-deficient males, T3.70^+^ thymocyte numbers remained lower in males with respect to females. The increase of thymocyte numbers correlated with reduced apoptosis in Rec-HY Bim-deficient male mice (**Figure 4C**).

T3.70^+^ CD8 T cells were found in the spleens of Rec-HY Bim WT and Bim deficient males. These cells had downregulated CD8 levels similar to the original H-Y TCR transgenic males. However, DN TCR^hi^ T cells, which are thought to be a result of aberrant development in the original H-Y TCR transgenic mouse [7], were not detected in any of the strains (**Figure 4D** **)**. Interestingly, a slight increase of T3.70^+^ CD4 lymphocytes was found in the Rec-HY Bim deficient male and female mice. The similar number of T3.70^+^ lymphocytes found in Rec-HY female and male mice (**Figure 4E**) might be related to the ability of H-Y T cells to undergo homeostatic proliferation in males in the absence of polyclonal T cells [33].

### Self-Reactive Bim-Deficient Thymocytes Have a CD4^lo^CD8^lo^ Phenotype

**Figure 5:**
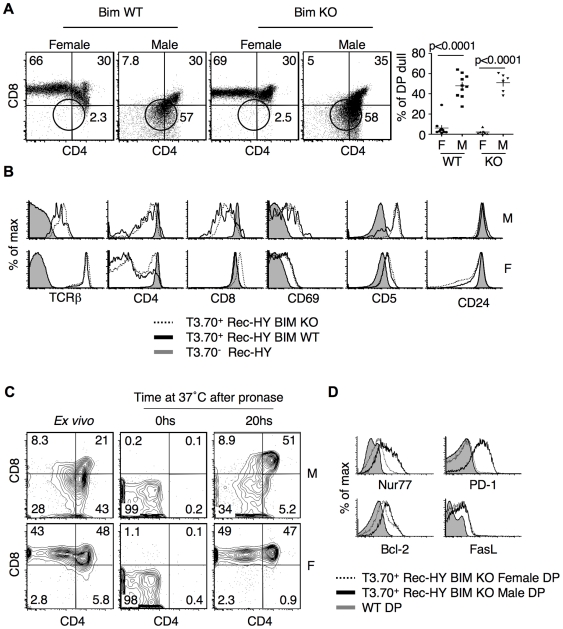
Bim-independent negative selection in Rec-HY males. **A**) FACS analysis of CD4 and CD8 on T3.70^+^ electronically gated thymocytes from Rec-HY Bim-sufficient (Bim WT) and Bim-deficient (Bim KO) female and male mice. The frequency of thymocytes inside the circular gate indicating the CD4^lo^CD8^lo^ phenotype is shown. Quantification of the percentage of thymocytes that acquired a CD4^lo^CD8^lo^ phenotype is showed for Bim wild-type (WT) and deficient (KO) Rec-HY female (F) and male (M) mice. (n = 10 for Bim WT mice, n = 6 for Bim KO mice the p values are represented in the graph). **B**) FACS analysis for markers expressed by HY-TCR positive thymocytes (T3.70^+^) from Rec-HY males (M) or females (F) comparing Bim-sufficient (Bim WT) with Bim-deficient (Bim KO) thymocytes. T3.70^−^ thymocytes from Rec-HY females are showed for comparison. **C**) FACS analysis for CD4 and CD8 expression on thymocytes before and after pronase digestion and re-expression cultures. Thymocytes were analyzed from Rec-HY Bim-deficient males (M) and females (F). The analysis shows T3.70^+^ and CD24^hi^ thymocytes. The numbers represent the frequency of events within each quadrant. **D**) Intracellular FACs analysis for Nur77, PD-1, Bcl-2 and FasL in electronically gated T3.70^+^ DP thymocytes from Rec-HY Bim-deficient males and females in comparison with wild type DP thymocytes. (A) is representative of more than six experiments, (B) is representative of four, (C) is representative of three and (D and E) are representative of two.

Although there was increased survival of thymocytes in Rec-HY Bim-deficient male mice, there was not an increased proportion of T3.70^+^ CD8 SP in the thymus. Rather there were simply increased numbers of thymocytes with an equivalent percentage of the cells at the CD4^lo^CD8^lo^ stage of development as in the Rec-HY Bim-sufficient male mice (**Figure 5A**). T3.70^+^ thymocytes in Rec-HY female mice, on the other hand, in both Bim-sufficient and Bim-deficient backgrounds differentiated towards the CD8 SP stage and were not found in the CD4^lo^CD8^lo^ population. T3.70^+^ thymocytes in Rec-HY Bim-sufficient and Rec-HY Bim-deficient backgrounds had similar expression levels of TCRβ, CD4, CD8, CD69, CD5 and CD24 (**Figure 5B**), suggesting that the CD4^lo^CD8^lo^ cells in the Rec-HY Bim-sufficient and Rec-HY Bim-deficient male mice were developmentally equivalent.

We next wanted to evaluate if T3.70^+^ CD4^lo^CD8^lo^ thymocytes from Rec-HY Bim-deficient male mice were committed to the CD8 SP lineage. For this purpose, pronase treatment and re-expression assays were carried out. Thymocytes treated with pronase were stripped of cell surface CD4 and CD8, but TCR and CD24 expression was retained. Re-expression of CD4 and CD8 after pronase stripping reveals the transcriptional status of these genes, which is indicative of the lineage commitment of the cells [34]. Total thymocytes from Rec-HY Bim-deficient male and female mice were treated with pronase followed by a twenty-hour incubation at 37°C. Immature T3.70^+^ CD24^hi^ thymocytes were identified before and after the pronase treatment. Cell surface CD4 and CD8 was absent immediately following the pronase treatment (**Figure 5C**). Following incubation, the immature cells from female mice had either a DP or CD8 SP phenotype at percentages similar to what was found prior to treatment. Approximately half of the clonotype positive thymocytes from male mice regained a DP phenotype following the incubation period. The remainder of the cells had a diffuse DN-DP double dull phenotype. This result indicated that cells undergoing negative selection were not transcribing high CD8 levels characteristic of commitment to the CD8 lineage.

We next analyzed the expression levels of molecules that have been related to thymocyte selection and apoptosis. T3.70^+^ Rec-HY Bim-deficient DP thymocytes from males had increased levels of Nur77 and PD-1, slightly increased Bcl-2, but the same levels of FasL when compared to T3.70^+^ Rec-HY Bim-deficient DP thymocytes from females (**Figure 5D**). Increased Nur77 levels were reported to be involved in the deletion of self-reactive thymocytes [11], [35], [36], raising the possibility that Nur77 might be responsible for the elimination of CD8 SP thymocytes in Rec-HY Bim-deficient males.

### Negatively Selected Thymocytes Accumulate Surrounding Medullar Areas in Rec-HY Bim-Deficient Males

**Figure 6:**
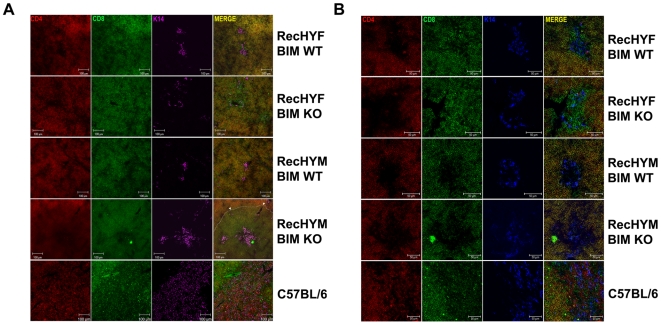
CD4^dim^ thymocytes localize surrounding medullar areas in Rec-HY Bim-deficient males. **A**) Confocal analysis of the thymus from C57BL/6, Rec-HY Bim-sufficient (Bim WT) and -deficient (Bim KO) female and male mice. Staining with anti-CD4 and anti-CD8 allows localization of SP and DP thymocytes relative to clusters of medullary thymic epithelial cells identified by staining with anti-K14 antibody. The dashed line indicated by white arrows indicates the accumulation of CD4^dim^ DP thymocytes. The horizontal white bar indicates a 100um magnification. **B**) Higher magnification for the same stainings represented in A. The horizontal white bar indicates a 50 um magnification. The staining with different antibodies is indicated by different colors in the figure. These results are representative of three independent experiments.

It is not clear where negative selection takes place in the thymus. The accumulation of CD4^lo^CD8^lo^ thymocyte in male Rec-HY Bim-deficient mice suggested that it might be possible to visualize cells undergoing negative selection in the thymus by microscopy. Although we were unable to detect T3.70^+^ thymocytes by microscopy, we used CD4, CD8 and Keratin14 (K14) markers to identify DP, SP thymocytes and medullary thymic epithelial cells. At low magnification, an accumulation of CD4^dim^ DP thymocytes was observed surrounding medullary areas in Rec-HY Bim-deficient males (dashed line, **Figure 6A**), indicating that in the absence of Bim this is the place where deletion takes place. At the cortico-medullary junction dendritic cells are responsible for the deletion of self-reactive thymocytes, suggesting that this process may occur independently of Bim. In Rec-HY Bim-sufficient males, however, we did not observe this accumulation of DP thymocytes, suggesting that Bim-dependent deletion occurred in the cortex. These data indicates that Bim-dependent and –independent deletion of self-reactive thymocytes occurs in different thymic compartments.

Some DP thymocytes with CD4^dim^ or CD8^dim^ staining were also observed in Rec-HY female and male mice scattered throughout the cortex. Analysis at higher magnification revealed an accumulation of CD8 SP thymocytes in medullary areas of Rec-HY BIM-sufficient and -deficient females but not males. As a control for the staining CD4 SP and CD8 SP thymocytes were clearly detected in wild type mice associated with medullary K14^+^ cells (**Figure 6B**).

### Ex Vivo Differentiation of Rec-HY Thymocytes

In male Rec-HY mice, a high percentage of Bim sufficient T3.70^+^ thymocytes are apoptotic and the cells appear to be rapidly eliminated. In the absence of Bim, few T3.70^+^ cells in male Rec-HY mice are apoptotic and the cells accumulate. The cells fail to develop to the CD8 SP stage and, therefore, still die. To further explore the mechanism of this delayed cell death, we collected CD24^hi^ T3.70^+^ DP thymocytes from Rec-HY Bim-deficient male and female mice by cell sorting, and placed them in culture with OP9–DL1 stromal cells, which are known to create an artificial environment that is capable of supporting early T cell development [37]. The cultures were supplemented with IL-7 to maintain viability of the cells. This differentiation assay could not be performed on sorted T3.70^+^ thymocytes from Rec-HY Bim-sufficient male mice, as they did not survive the culture (data not shown).

**Figure 7:**
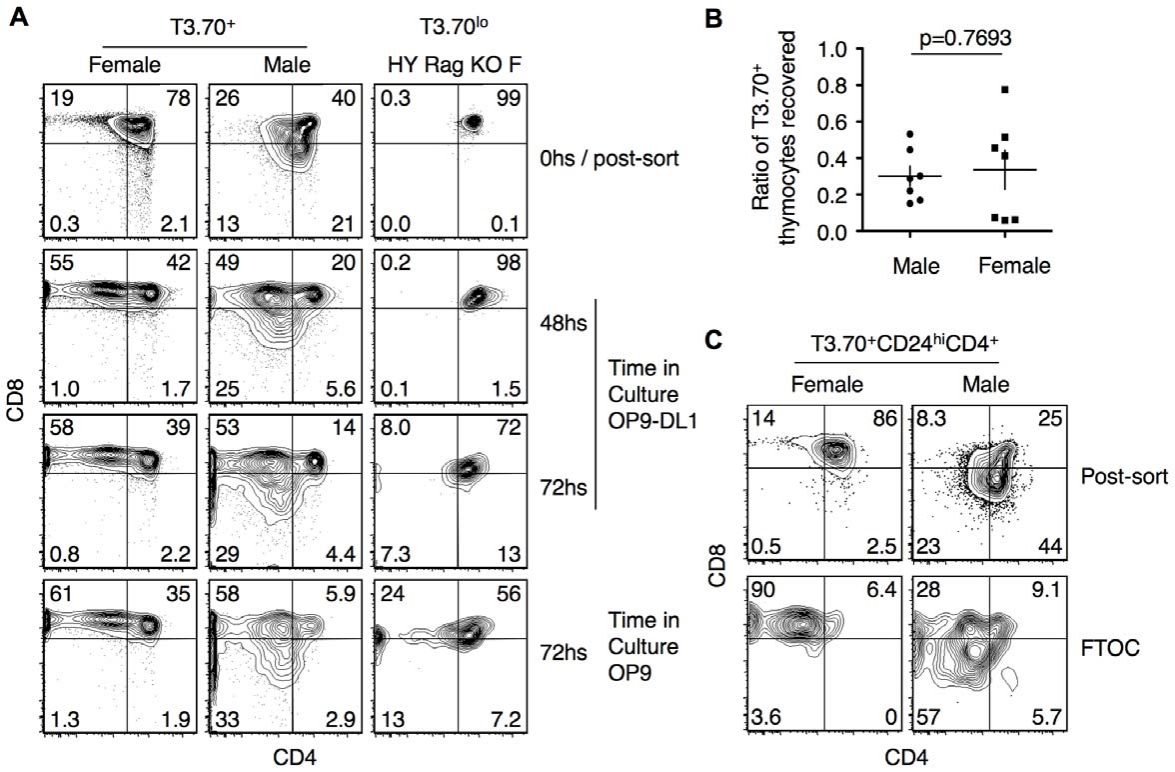
Autoreactive Self-reactive DP thymocytes can differentiate in vitro. **A**) CD4 vs CD8 contour plotFACS analysis of sorted T3.70^+^CD4^+^CD24^+^ thymocytes from Rec-HY BimBim-deficient females and males or T3.70^lo^CD4^+^CD24^+^ thymocytes from HY-TCR Rag2-deficient female mice (0 hsHY Rag KO F), after post-sort or 48 and 72 hs of co-culture with OP9-dl1 DL1 or OP9-GFP stromal cell in the presence of IL-7.s. The numbers represent the frequency of events within each quadrant. **B**) The total number of live (DAPI negative) thymocytes that was recovered after 48 hs co-culture with OP9-DL1 cells in the presence of IL-7 was divided by the number of cells seeded for each experiment. The horizontal and vertical bars represent the average and SEM respectively (n = 7). The p value, indicated in the Figure, did not reach significance. **C**) FACS analysis of sorted T3.70^+^CD4^+^CD24^+^ from Rec-HY Bim-deficient females or males. Thymocytes were immediately stained after sort (Post-sort) or analyzed 48 hs after seeding and incubation in 2-deoxyguanosine treated FTOCs derived from C57BL/6 fetuses. The numbers represent the frequency of events within each quadrant. (A and B) are representative of seven independent experiments and (C) is representative of two.

Sorted cells were stained for CD4 and CD8 expression immediately after sorting and then again 48 and 72 hours after being placed in culture. Rec-HY thymocytes from female mice acquired a CD8 SP phenotype after 48 hours in culture (**Figure 7A**). Remarkably, Rec-HY thymocytes from male mice also acquired a CD8 SP phenotype. An additional 24 hours in culture did not promote further development. OP9 stromal cells not transfected with delta-1 also supported the differentiation of the clonotypic thymocytes and, therefore, notch-mediated signals did not influence development (**Figure 7A**). Importantly, sorted clonotype negative DP thymocytes from Rec-HY Bim-deficient mice (data not shown) or thymocytes from conventional HY TCR H-Y TCR transgenic Rag-deficient females expressing low HY-TCR levels were not able to differentiate *ex vivo* (**Figure 7A**). These controls showed that *ex vivo* differentiation was triggered by the TCR signals received *in vivo* and not by staining with the T3.70 antibody. The proportion of thymocytes recovered after the 48hs incubation in OP9-DL1 co-cultures was not significantly different between male and female thymocytes (**Figure 7B**). Next, sorted thymocytes transfer into 2-deoxyguanosine treated fetal thymic lobes and incubated for 48 hours (**Figure 7C**). Importantly, it was confirmed that, unlike in conventional FTOCs, the high oxygen conditions that were used for seeding of thymic lobes allowed for wild type DP thymocytes to penetrate the fetal thymic lobes 24 hs after seeding (data not shown). Again, as seen for the OP9 cultures, both double positive from either females or males clearly had the potential to develop to a CD8 single positive-like stage. Differentiation was, however, more efficient for female derived than male derived cells. Overall, these results showed that self-reactive clonotype thymocytes were able to initiate differentiation towards CD8 SP once removed from the adult thymus.

### Bim-Independent Deletion of T3.70^+^ Thymocytes in H-Y-TCRβ Transgenic Males

**Figure 8:**
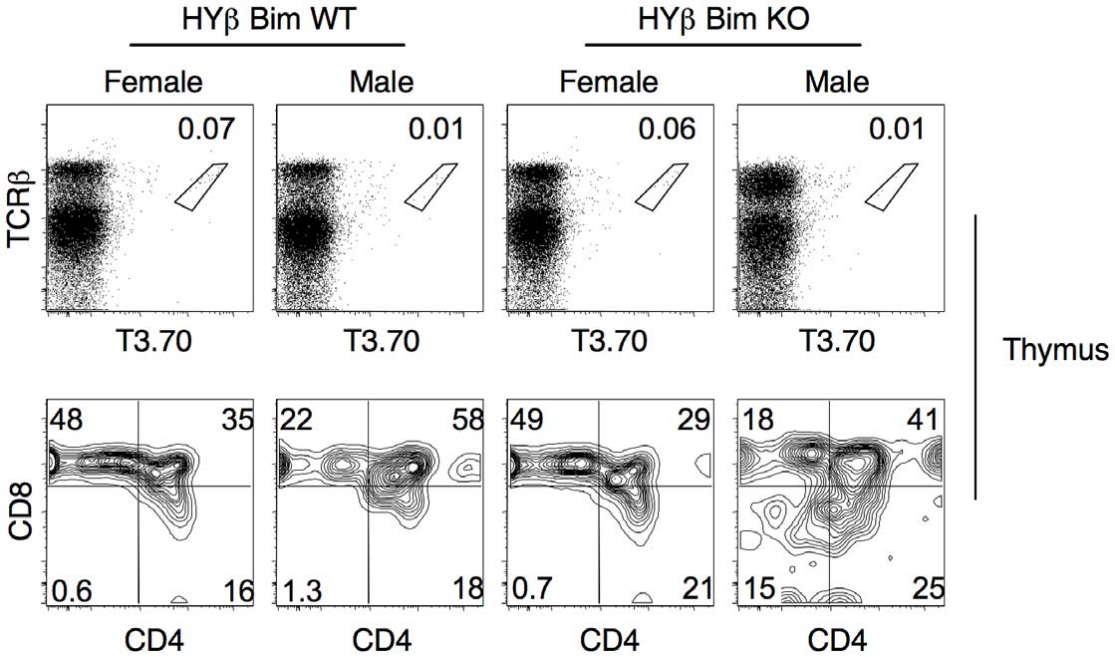
Bim-independent negative selection in H-Y TCRβ transgenic mice. FACS analysis of thymocytes from H-Y TCRβ transgenic (HYβ) males and females in Bim-sufficient (Bim WT) and Bim-deficient (Bim KO) backgrounds. CD4 and CD8 profiles of T3.70^+^ electronically gated thymocytes. The frequency of thymocytes corresponding to each gate is represented within the graphs. These results are representative of two independent experiments.

Next we analyzed the fate of negatively selected, Bim-deficient thymocytes in a second TCR transgenic mouse model. In mice carrying the transgene only for the TCRβ chain, the TCRα chain is derived from the endogenous locus. Partially restricting TCR diversity by this means often produces an increase in the frequency of some TCR specificities [38], [39]. Therefore, we analyzed female mice carrying only the H-Y TCRβ transgene by FACS to determine if T3.70^+^ thymocytes could be detected. A small, but distinct population of T3.70^+^ thymocytes was clearly detected in female H-Y TCRβ transgenic mice (**Figure 8**). This population was not detected in non-transgenic littermates (data not shown). By single cell sequencing we determined that the clonotype positive population of cells has TCRα chains that are identical or similar to the H-Y TCRα chain (Stolzer and Sant'Angelo, unpublished). Importantly, the clonotype positive thymocytes were strongly skewed towards the CD8 lineage, similarly to T3.70^+^ thymocytes from Rec-HY females. Interestingly, the population of T3.70^+^ thymocytes in H-Y TCRβ transgenic males was reduced by more than 80%, suggesting that in males a large proportion of the T3.70^+^ cells were undergoing negative selection. The residual T3.70^+^ cells have TCRs that are distinct from the HY TCRα chain (Stolzer and Sant'Angelo, unpublished).

Bim-deficient H-Y TCR TCRβ transgenic mice were generated to study the impact of loss of Bim in this second model of negative selection. Bim deficiency in H-Y TCRβ transgenic males did not rescue the negatively selected T3.70^+^ thymocytes (**Figure 8**). Loss of Bim did, however, lead to an increase of T3.70^+^ thymocytes with the CD4^lo^CD8^lo^ phenotype that was observed in the Rec-HY mice (**Figure 8**).

Overall, our data support a model in which negatively selected Bim-sufficient self-reactive thymocytes enter a CD4^lo^CD8^lo^ stage of development and are rapidly eliminated. In the absence of Bim, negatively selected cells still obtain the CD4^lo^CD8^lo^ phenotype, but there is a significant delay before death. Finally, the Bim-independent pathway for negative selection is dependent upon the intact adult thymic microenvironment. Overall, these data newly reveal a layering of pathways that contribute to elimination of self-reactive thymocytes in vivo.

## Discussion

We have used a novel recombination-dependent TCR transgenic mouse model (Rec-HY) to study the process of negative selection in the thymus. In these mice, the expression of the H-Y TCR was dependent on the machinery that normally directs V(D)J recombination of the endogenous TCRα locus. As a result, both the timing and levels of the TCR closely mimic the endogenous locus. Furthermore, our system also mimics the wild type thymic environment by producing DP thymocytes that are unable to productively interact with MHC and, therefore, die by neglect. Even total thymocyte numbers in Rec-HY, which are mainly influenced by pre-selected DP thymocytes, were, as a result, similar to wild-type mice. Conventional transgenes, on the other hand, cause expression of the TCRα chain very early in development and at artificially high levels [40]. Ectopic expression of the TCRα chain dramatically disrupts T cell development, resulting in reduced cell numbers and aberrant T cell populations. To bypass the early TCR expression problem, one group has produced a model in which the TCRα chain is not expressed until the DP stage of development [5]. Multiple lines of evidence, however, show that normal expression of the TCRα chain precedes the DP stage of development, suggesting that this model is also problematic.

The Rec-HY TCR transgenic model allowed us to clearly identify and study self-reactive thymocytes that have rearranged their TCR but have not yet been deleted. Consistent with having received stronger TCR signals, self-reactive thymocytes had increased levels of CD69 and CD5 and lower levels of TCR, CD4 and CD8. This CD4^lo^CD8^lo^ phenotype is demonstrably different than the CD69^+^CD4^lo^CD8^lo^ population of cells that develops immediately post-positive selection [24], [25]. In particular, the self-reactive cells had much lower levels of CD8 expression and most of the T3.70^+^ thymocytes in Rec-HY males were dying by apoptosis. Self-reactive thymocytes also expressed Nur77 and Bim, which have been correlated with the apoptosis of DP thymocytes during negative selection [11], [12].

Using our model system, we reevaluated the requirement for Bim in the apoptosis and negative selection of self-reactive thymocytes. Bim-deficiency clearly reduced the level of apoptosis of self-reactive thymocytes. The negatively selected cells, however, were found to accumulate at the immature CD4^lo^CD8^lo^ stage of development rather than progress to the mature single positive stage of development, as suggested by a previous report [41]. Pronase stripping/re-expression assays showed that the self-reactive thymocytes actively transcribed low levels of both CD8 and CD4 indicating that the developmental stage of these cells was at a point prior to downregulation of CD4 and upregulation of CD8. Although apoptosis was reduced by Bim-deficiency, the numbers of cells within the CD4^lo^CD8^lo^ population in the negatively selection male environment was still significantly lower than the numbers of positively selected thymocytes in Rec-HY females. These data clearly shown that deletion of self-reactive thymocytes can also occur independently of Bim.

Microscopic analysis of the thymus revealed an accumulation of CD8^+^CD4^dim^ thymocytes surrounding medullar areas in Rec-HY Bim-deficient males, but not in Rec-HY Bim-deficient females. Also, the accumulation of these cells was not observed in Bim-sufficient males. This result is indicative of the two different mechanisms we propose are in effect for the deletion of self-reactive thymocytes. Our data suggest that there is a Bim- dependent mechanism that occurs essentially simultaneously with the initial TCR signal, while a second Bim-independent mechanism is delayed, allowing thymocytes to accumulate in regions surrounding the medulla.

It was surprising to observe that the increased survival and accumulation of self-reactive thymocytes in Bim-deficient male mice did not correlate with increased differentiation. In particular, because these cells expressed markers such as CD69 and CD5, which have are associated with positive selection. This prompted us to question if the failure of self-reactive CD4^lo^CD8^lo^ thymocytes in Rec-HY Bim-deficient mice to progress to a mature single positive stage of development was dependent on the negatively selecting thymic environment. To test this possibility, CD4^lo^CD8^lo^ thymocytes from Rec-HY Bim-deficient male mice were transfered into *in vitro* systems of T cell development. Effectively, we observed that self-reactive CD4^lo^CD8^lo^ thymocytes were able to differentiate after transfer to OP9-dl1 or FTOCs cultures.

It is possible that deletion of thymocytes *in vivo,* in the absence of Bim, is mediated by the elevated Nur77 levels. It was recently suggested that the pro-apoptotic function of Nur77 in thymocytes is mediated by migration of Nur77 to the mitocondria changing the function of Bcl-2 by exposing its BH3-domain [42]. However, TCR stimulation of both T cells and immature thymocytes lead to increased Nur77 levels, but only in thymocytes it triggers apoptosis. This different outcome has been correlated to different posttranslational modifications of the protein [43]. Consistent with this possibility, we showed that self-reactive CD4^lo^CD8^lo^ Bim-deficient thymocytes have high Nur77 levels. However, as discussed above, in vitro, the CD4^lo^CD8^lo^ Bim-deficient thymocytes progress to the CD8SP stage of development. These results suggest that, although Nur77 might substitute for Bim in vivo, Nur77 is not sufficient to mediate cell death outside of the thymic microenvironment.

Another possible explanation for the recovery of differentiation that we observed *in vitro* is that the cells that mediate Bim-dependent and –independent deletion of thymocytes are different. For example, it has been shown using the “on-time” H-Y TCR transgenic model that deletion of thymocytes can occur in the cortex without involvement of the medulla [3], [4]. We were not able to localize self-reactive thymocytes in the Bim-sufficient strains, suggesting that negative selection occurred throughout the thymus, including the cortex. Bim-deficient self-reactive thymocytes, however, accumulated around the medullary regions. At this site, deletion of self-reactive thymocytes can be mediated by dendritic cells [2]. Professional antigen presenting cells, however, were not present in the OP9-dl1 cultures or in 2-deoxyguanosine-pretreated FTOCs, which may explain the differentiation of thymocytes *in vitro* but not *in vivo*.

It was previously shown than Bim deficiency prevents apoptosis of DP thymocytes in the original H-Y TCR transgenic male mice [12]. In agreement with this work, our results support a role for Bim in the apoptosis of thymocytes during negative selection. Reduction of apoptosis by Bim deficiency, however, didn't lead to increased T3.70^+^ CD8 SP thymocytes in Rec-HY males. This discrepancy is likely due to the aberrant differentiation that occurs in the original H-Y TCR model as consequence of the overexpression of the transgene. Indeed, the high levels of expression of the H-Y transgene early in T cell development have recently been shown to cause the diversion of H-Y TCR expressing cells into DN and CD8αα γδ-like T cells bypassing the DP developmental stage [7].

TCRβ transgenic mice are a highly physiological model, in which the diversity of the TCRα repertoire is restricted as a consequence of positive selection [25]. This restriction allows the detection of specific TCRα rearrangements in the naïve T cell population that would not be possible in normal mice. We detected a small proportion of clonotype T3.70^+^ thymocytes in H-Y TCRβ transgenic females, which indicates that some TCRα rearrangements were recognized by the T3.70^+^ antibody. T3.70^+^ thymocytes in H-Y TCRβ transgenic females represent a small population of cells containing different TCRα chains (Stolzer and Sant'Angelo unpublished). In H-Y TCRβ transgenic males, the proportion of T3.70^+^ thymocytes was highly reduced, which was indicative of negative selection of most of these cells. Similar to what was found in the Rec-HY system, Bim deficiency did not rescue the reduced frequency of T3.70^+^ thymocytes in H-Y TCRβ males with respect to females. Therefore, in a second model, negative selection still occurs in the absence of Bim.

In conclusion, we have developed a novel V(D)J recombination dependent transgene that closely mimics expression of the endogenous TCR locus. With this model, negatively selected thymocytes can be identified prior to death in a highly physiological setting, allowing the study of their phenotypic characteristics. We find that negatively selected cells accumulate with an activated, CD4^lo^CD8^lo^ phenotype. Next we used this model, to further study the role of the pro-apoptotic protein, Bim during negative selection. We found that in the absence of Bim negatively selected thymocytes accumulated with the activated, CD4^lo^CD8^lo^ phenotype, but, intriguingly, differentiation was still impaired. Removal of the CD4^lo^CD8^lo^ thymocytes from the negatively selecting environment, however, allowed for continued development. Therefore, Bim appears to control the rapid apoptosis of self-reactive thymocytes, whereas Nur77 likely controls a secondary, delayed apoptosis that is dependent on the thymic microenvironment. We speculate that chronic TCR signaling is likely involved in the Nur77 dependent negative selection. Overall, our data show that negative selection can be accomplished both by a fast, Bim-dependent pathway and also by a slower Bim-independent pathway that is wholly dependent upon an intact thymic microenvironment. Therefore, different and independent mechanisms converge for the deletion of self-reactive thymocytes.

## Materials and Methods

### Mice

Construction of the Rec-HY transgene was performed by insertion of the rearranged H-Y TCRα coding sequence into the pTαCass transgene vector [14] with a reconstituted complete TCRα LCR [15]. The start codon from the Rec-HY TCRα transgene was separated from the rest of the V-J-Cα coding sequence by a 5 KB “stuffer” DNA fragment containing the RSS recombination sequences. Rec-HYα transgenic mice were crossed to H-Y TCRβ mice, which were generated by the microinjection of the DNA construct used for the original H-Y TCR transgenic mice [13], and TCR Cα deficient mice. C57BL/6 and Bim-deficient mice where purchased from Jackson Laboratories. Mice were used between one and two months of age. All animal work was done in compliance with MSKCC's IACUC. Compound H-Y TCRβ Bim-deficient mice were generated by breeding the two strains.

### Flow Cytometry

Surface staining was performed in FACS buffer (PBS with 2% heat inactivated FBS) for 20 min on ice using the indicated surface antibodies from BD Biosciences (San Jose, CA), e-biosciences (San Diego, CA), or from the MSKCC Antibody Core Facility. Data acquisition was performed on an LSRII cytometer BD Biosciences (San Jose, CA) and exclusion of dead cells was performed by DAPI staining. Cell doublets were removed by monitoring the pulse width channel. Data was analyzed using the FlowJo software (TreeStar Inc., Ashland, OR). Fixation and permeabilization for intracellular staining was performed using the e-bioscience (San Diego, CA) intracellular kit.

### Antibody Clones

Anti-CD4 (RM4-5), anti-CD8α (53.6.7), anti-CD8β (H35-17.2.), anti-CD24 (M1/69), anti-TCRβ (H57.597), anti-CD5 (53-7.3), anti-CD69 (H1.2F3), anti-HY-TCR (T3.70). For the analysis of the DN subsets, exclusion of lineage positive cells was performed by staining with anti-CD19 (1D3), anti-γδTCR (GL3), anti-Ter119 (TER119), anti-CD49b (DX5) anti-NK1.1 (PK136), anti-GR1 (RB6-8C5), anti-CD11b (M1/70) and the different subsets were identified according to the CD25 (PC61) and CD44 (IM7) levels. Detection of membrane TCRα (Vα2) was performed with the anti-Vα2 (B20.1) antibody. Antibodies were used with different fluorochrome conjugations FITC-, PE-, PERCP5.5-, PEcy7-, APC- and APCcy7. Intracellular staining for Bim (polyclonal rabbit) (Cell signaling tech, Danvers, MA) was performed using the eBioscience Intracellular staining kit (San Diego, CA), according to the manufacture's instructions.

### Pronase Assays

Pronase digestion was performed on total thymocytes from Rec-HY Bim-deficient mice. Briefly, cells were resuspended in 1 ml of Pronase buffer (0.04% pronase, 100 ug/ml DNAseI in PBS) and incubated 10 min at 37°C. Immediately after, cells were diluted 20 times in ice cold media with 20% serum and washed twice in complete IMDM with 20% FBS. Immediately, or after the indicated times of incubation at 37°C, thymocytes were surface stained and analyzed by FACS.

### Confocal Microscopy

Frozen cryostat sections (8 mm) were prepared, air dried and fixed with 4% paraformaldehyde for 20 min at RT, followed by three washes with PBS for 5 min. Fixed sections were incubated with anti-CD4 PE (GK1.5), anti CD8 FITC (53–6.7) (BD Biosciences) and polyclonal rabbit anti-Keratin 14 (Covance) diluted in staining buffer (SB) (1× PBS containing 5% normal donkey serum, 0.1% TritonX-100 and 1% BSA) for 1 hr at 37°C. Sections were washed three times with PBS for 5 min prior to incubation with CY5-conjugated donkey anti-Rabbit IgG secondary antibody (Jackson) diluted 1∶200 in SB for 20 min at RT. Slides were again subjected to three washes with PBS for 5 min. Sections were mounted in ProLong Gold anti-fade reagent with DAPI (Molecular Probes) and observed using a Zeiss 510 confocal microscope or a Nikon TE2000 inverted microscope equipped with epifluorescence and a SPOT digital camera system.

### Annexin V Staining

Apoptotic cells were detected using the Annexin V staining protocol (BD Biosciences, San Jose, CA) according to the manufacturer's instructions. After surface staining of thymocyte suspensions cells were washed and resuspended in 100 ul of Annexin V binding buffer containing 5 ul of Annexin V-FITC. Cells were incubated for 15 min at room temperature, diluted in Annexin V binding buffer containing DAPI and immediately analyzed by FACS.

### OP9-DL1 Cultures

Sorted thymocytes were placed in co-culture with OP9-dl1 or OP9-GFP stromal cells in the presence of 1 ng/ml of IL-7 Peprotech (Rocky Hill, NJ) for 48 or 72 hs. After culture, thymocytes were re-stained for surface markers and analyzed by FACS.

### FTOC Reconstitution

15.5 day-old fetal thymi was incubated for 6 days in 200 ml RPMI1640 complete media (10% FBS and 1.35 mM 2-deoxyguanosine) to eliminate thymocytes. FTOCs cultures were performed in V-bottom 96-well plates (BD) in a high oxygen (70% O_2_, 25% N_2_, 5% C0_2_) chamber (BioSpherix C-Chamber fitted with ProOx and ProCO2 controllers) at 37°C. After 6 days, thymi were washed in 10% complete RPMI and sorted thymocytes were added to the FTOCs. The plates were centrifuged at 1000xG for 3 min to promote thymocyte contact with the lobes. After 48 hrs in high oxygen culture the lobes were removed, washed extensively and analysis of thymocytes was performed by FACS.

### Semiquantitative RT-PCR

cDNA was prepared by reverse transcription using the Superscript II RT kit (Invitrogen, San Diego, CA). After equilibration of cDNA samples based on actin levels, serial 1∶5 dilutions of the target DNA was performed and specific PCRs for the indicated genes were analyzed.

### Statistical Analysis

Statistical analysis was performed using the Prism software, all samples were analyzed using the unpaired and 2-tail T-test.

## Supporting Information

Figure S1 Expression of markers in CD8 SP and CD8lo SP in Rec-HY females and males. FACS analysis showing membrane CD4 vs CD8 from T3.70+ thymocytes in Rec-HY female and male mice. The events within the indicated CD8 SP gate were used for comparison of marker expression represented in the histograms. This experiment is representative of four.
(0.19 MB TIF)
