# Supplementary figures and images for: A Novel TCR Transgenic Model Reveals That Negative Selection Involves an Immediate, Bim-Dependent Pathway and a Delayed, Bim-Independent Pathway
Source: PLoS One. 2010 Jan 13;5(1):e8675. doi: 10.1371/journal.pone.0008675 (PMC2800196; doi:10.1371/journal.pone.0008675)

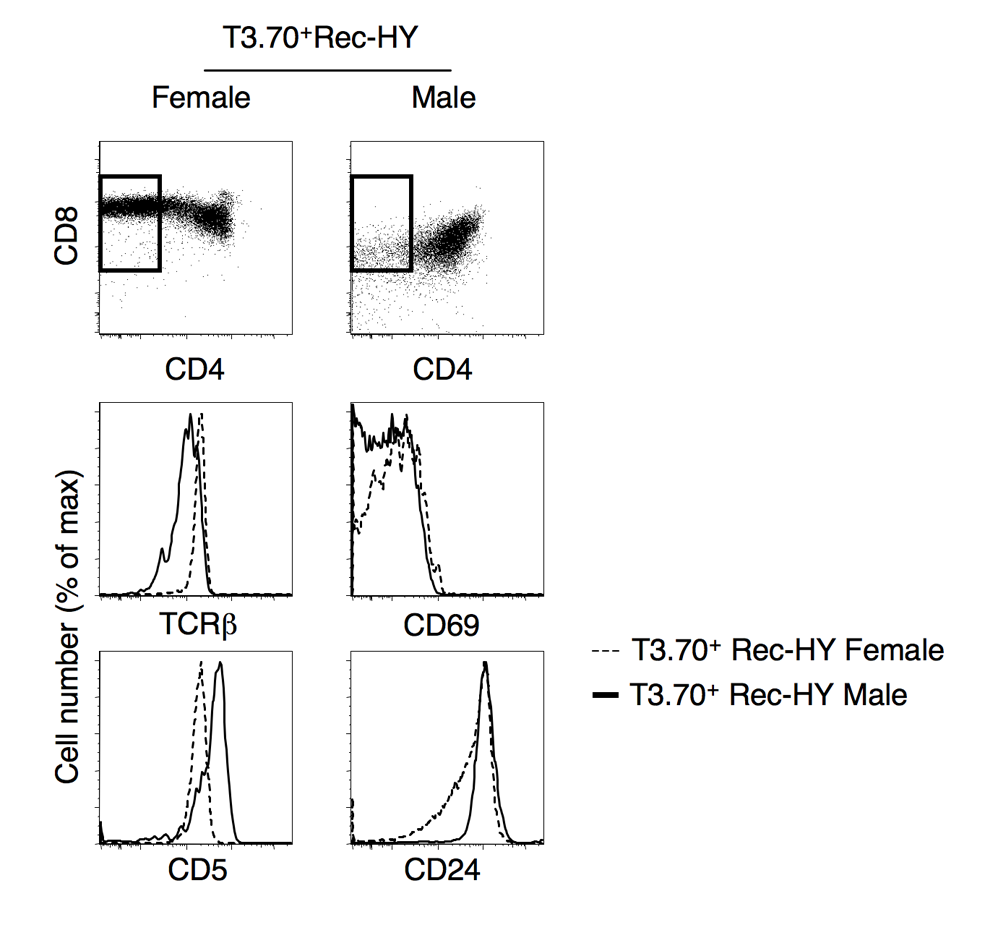

Supplement: Figure S1 — Expression of markers in CD8 SP and CD8lo SP in Rec-HY females and males. FACS analysis showing membrane CD4 vs CD8 from T3.70+ thymocytes in Rec-HY female and male mice. The events within the indicated CD8 SP gate were used for comparison of marker expression represented in the histograms. This experiment is representative of four. (0.19 MB TIF) [file pone.0008675.s001.tif]
